# Feasibility and efficacy of text messaging to promote care among trauma patients screened for HIV at an urban emergency department in Tanzania

**DOI:** 10.1186/s12245-021-00395-0

**Published:** 2021-12-14

**Authors:** Gimbo M. Hyuha, Hendry R. Sawe, Said Kilindimo, Raya Y. Mussa, Masuma A. Gulamhussein, Shamila S. Rwegoshora, Frida Shayo, Winnie Mdundo, Juma A. Mfinanga, Ellen J. Weber

**Affiliations:** 1 Emergency Medicine Department, Muhimbili University of Health and Allied Sciences, P.O. Box 65001, Dar es Salaam, Tanzania; 2 Emergency Medicine Department, Muhimbili National Hospital, Dar es Salaam, Tanzania; 3 Department of Emergency Medicine, University of California San Francisco, San Francisco, CA USA

**Keywords:** HIV, AIDS, SMS, Text message, Reminder, Cell phone, Opt-out, PITC, Trauma, Emergency department, Tanzania

## Abstract

**Background:**

Due to the high prevalence of human immunodeficiency virus (HIV) in Tanzania, provider-initiated HIV testing for patients attending any health care setting is recommended. However, follow-up and linkage to care by those tested remain poor. We determined the feasibility and efficacy of text messaging to promote follow-up among otherwise healthy trauma patients who underwent provider-initiated HIV testing and counseling at an emergency department (ED) in Tanzania.

**Material and methods:**

This randomized controlled trial (RCT) was conducted at Muhimbili National Hospital (MNH) ED between September 2019 and February 2020. Adult trauma patients consenting to HIV testing and follow-up text messaging were randomized to standard care (pre-test and post-test counseling) or standard care plus a series of three short message service (SMS) text message reminders for follow-up in an HIV clinic, if positive, or for retesting, if negative. Investigators blinded to the study assignment called participants 2 months after the ED visit if HIV-positive or 4 months if HIV-negative. We compared the proportion of people in the intervention and control groups completing recommended follow-up. Secondary outcomes were the proportion of patients agreeing to testing, proportion of patients agreeing to receiving text messages, and the proportion of HIV-positive and HIV-negative patients in each study arm who followed up.

**Results:**

Of the 290 patients approached, 255 (87.9%) opted-in for testing and agreed to receive a text message. The median age of the study population was 29 [IQR 24–40] years. There were 127 patients randomized to the intervention group and 128 to the control group. The automated SMS system verified that 381 text messages in total were successfully sent. We traced 242 (94.9%) participants: 124 (51.2%) in the intervention group and 18 (488%) in the control group. A total of 100 (39.2%) subjects reported completing a follow-up visit, of which 77 (60.6%) were from the intervention group and 23 (17.9%) were from the control group (RR = 3.4, 95% CI 2.3–5.0). This resulted in a number needed to treat (NNT) of 2.3. Of the 246 HIV-negative participants, 37% underwent repeat screening: 59% of those in the intervention group and 16% in the control group (RR = 3.7, *P* = < 0.0001, NNT 2.3). Among the nine positive patients, all five in the intervention group and only three in the controls had follow-up visits.

**Conclusion:**

Automated text message is a feasible and effective way to increase follow-up in HIV-tested individuals in a limited income country.

## Background

HIV continues to be a major global public health problem, claiming more than 35 million lives thus far. In 2017, 940,000 people died from HIV-related causes globally [1]. Sub-Saharan Africa, including Tanzania, is the most affected region in the world, with 25.8 million people living with HIV in 2017. Two-thirds of the global total of new HIV infections occur in sub-Saharan Africa [2]. In 2016, 1.4 million people were living with HIV in Tanzania. This equates to an estimated HIV prevalence of 4.7% [3].

Routine provider-initiated testing, with the opportunity for patients to opt-out, has been shown to identify people living with HIV/AIDS earlier, enroll them into care, and reduce mortality [4–6]. In Tanzania, the National Comprehensive Guideline for HIV Testing and Counseling of 2017 recommends routine testing and screening for HIV/AIDS in all health centers [6]. However, follow-up is poor in these patients after testing [5, 7, 8]. Failure to attend medical appointments among persons living with HIV has been associated with poor health outcomes [9, 10].

Text messaging via SMS is a potential intervention for improving retention and compliance with recommended care, by providing medication and appointment reminders [7, 8, 11–17]. Studies on the feasibility and efficacy of using text messaging via SMS in promoting care have been performed in many countries; almost all have shown an improvement in health care behaviors and disease outcomes [7, 8, 11–17]. Nevertheless, there are only a few studies on the feasibility and efficacy of using SMS as a means to promote care in sub-Saharan Africa and none in Tanzania [7, 8, 15]. Further, unlike high-income countries (HICs) where individuals frequently encounter the health care system due to established primary care systems, in low- and middle-income countries (LMICs), many people with HIV do not know they have it and will not be tested. The ability of text messaging to improve compliance among otherwise asymptomatic patients or those that need repeat screening has not been studied in any country.

The Muhimbili National Hospital - Emergency Department (MNH-ED) created a protocol for rapid HIV testing of trauma patients in 2018. Trauma patients are usually healthy and would not otherwise seek health care where provider-initiated HIV testing might occur. A pilot study in our ED found that these patients were willing to be tested, but of those who were HIV-positive, only half sought follow-up care [6]. We therefore tested the feasibility and efficacy of using automated text messaging to promote follow-up care among otherwise healthy patients screened for HIV at the MNH-ED.

## Methodology

### Study design

This was a single-center, randomized, blinded controlled trial of adult trauma patients presenting to the Emergency Department of Muhimbili National Hospital (MNH-ED) between September 2019 and February 2020 who agreed to provider-initiated, rapid HIV testing.

### Study setting

Muhimbili National Hospital (MNH) is a tertiary referral hospital with a level 1 trauma center located in Dar es Salaam, Tanzania. The hospital has a bed capacity of 1500, averaging 1000 to 1200 admissions per week. The MNH-ED is the first public emergency department (ED) in the country and opened in 2010 [18]. It is staffed with emergency physicians, postgraduate students in an emergency medicine training program, medical officers, critical care nurses, and nursing officers. The MNH-ED is the entry point to the hospital for most of the patients attending MNH and sees an average of 200 patients daily, with an admission rate of 65%. Acutely ill patients are received at the ED, resuscitated, and stabilized before being transferred to the appropriate ward.

### Participants

All adult patients with trauma attending the MNH-ED were eligible for the study. To be included, patients had to agree to be tested for HIV and be willing and able to receive text messages on their mobile phones. Patients were excluded if they were below 18 years old, had a Glasgow Coma Scale score below 15, known to be HIV-positive with evidence of either a treatment card or medications, were clinically unstable, did not own a cell phone or phone was not capable of receiving an SMS, or had been previously enrolled in the study.

### Study protocol

The study was registered in the Pan African Clinical Trials Registry (PACTR), with the trial registration number PACTR201909858930669. Researchers actively walked through the ED to identify trauma patients and used the tracking board of the ED’s electronic medical record (Wellsoft™): to identify patients triaged with a complaint related to trauma. Researchers screened patients for eligibility and willingness to have an HIV test and receive text messages. They then consented the patients to the study. Research assistants captured baseline patient data using a structured case report form; data were then transferred to an online data storage platform (Redcap™). A trained nurse provided pre-test and post-test counseling to all patients tested for HIV. Standard trauma treatment for injuries was performed at the discretion of the treating physician.

### Randomization and blinding

Prior to beginning enrollment, we created a computer-generated randomization scheme that ensured equal numbers of participants in the intervention and control groups [19]. Immediately after consent, patients were randomly assigned to the intervention or control group. Allocation was concealed from patients and research assistants; research assistants knew that the patient was assigned to group A or B but did not know which was the control or intervention group.

### Intervention

We used an automated Short Message Service (SMS) system to send texts at scheduled times to the patients in the intervention arm. Patient information and their group assignment were transferred to the Extrateq™-automated SMS dispatch program. SMS messages were sent only to patients that had been randomized to the intervention arm of the study. The program was set to deliver text messages three times for everyone in the intervention group, once per week for HIV-positive patients, and once per month for HIV-negative patients, starting from the day of the ED visit. SMS content was sent in Swahili with an English translation reading: “Dear EMD client, this is a reminder that it is important for your health that you visit the nearest hospital to you., Thank you in advance and have a good day.” To assess the fidelity of the intervention, automated SMS system was queried at the end of the study to determine how many texts were successfully sent.

### Patient follow-up

Follow-up phone calls were made by research assistants or the primary investigator 2 months after the ED visit if the subject was HIV-positive or months after the visit if the patient was HIV-negative. The researchers making the calls were blinded to the arm of the study the patient. All traced participants were asked during the follow-up phone call whether they received a message at least once and to report whether they attended their follow-up Care and Treatment Centre (CTC) clinic (if HIV-positive) or went for repeat HIV screening (if HIV-negative). Those who were not traced were considered lost to follow-up. Follow-up interview data was entered into the patient’s initial questionnaire and subsequently uploaded to the data storage platform.

### Outcomes

The primary outcome was the proportion of patients who reported attending follow-up visits either to the CTC clinic (HIV-positive) or another site for repeat HIV testing (HIV-negative). Based on a previous study by Mugo et al. [8] where compliance in the intervention and control groups was 59% and 41%, respectively, we determined the minimum sample size required was 240 (120 in each group). Secondary outcomes were the proportion of patients agreeing to testing, the proportion of patients agreeing to receive text messages, and the proportion of HIV-positive and HIV-negative patients in each study arm who followed up.

### Data analysis

Data was coded and imported into Research Electronic Data Capture (REDCap). Statistical Package for Social Science (IBM SPSS version 25, IBM, LTD, Carolina, USA) was used for analysis. Relevant frequencies and tables were generated for all variables. The means, proportions, medians, and interquartile range were calculated for continuous variables according to their distribution. An intent-to-treat analysis was used to arrive at our results.

The primary outcome was analyzed using Pearson’s chi-square test and the difference shown using relative risks (RR) with 95% confidence intervals (CI) and number needed to treat (NNT). The secondary outcomes were compared using Pearson’s chi-square test or Fisher’s exact test, and proportions or means were used to report secondary outcomes and differences shown by RR. Statistical analysis was two-tailed, and a *P* value of less than 0.05 was considered statistically significant.

## Results

### Recruitment and baseline characteristics of the study population

**Fig. 1:**
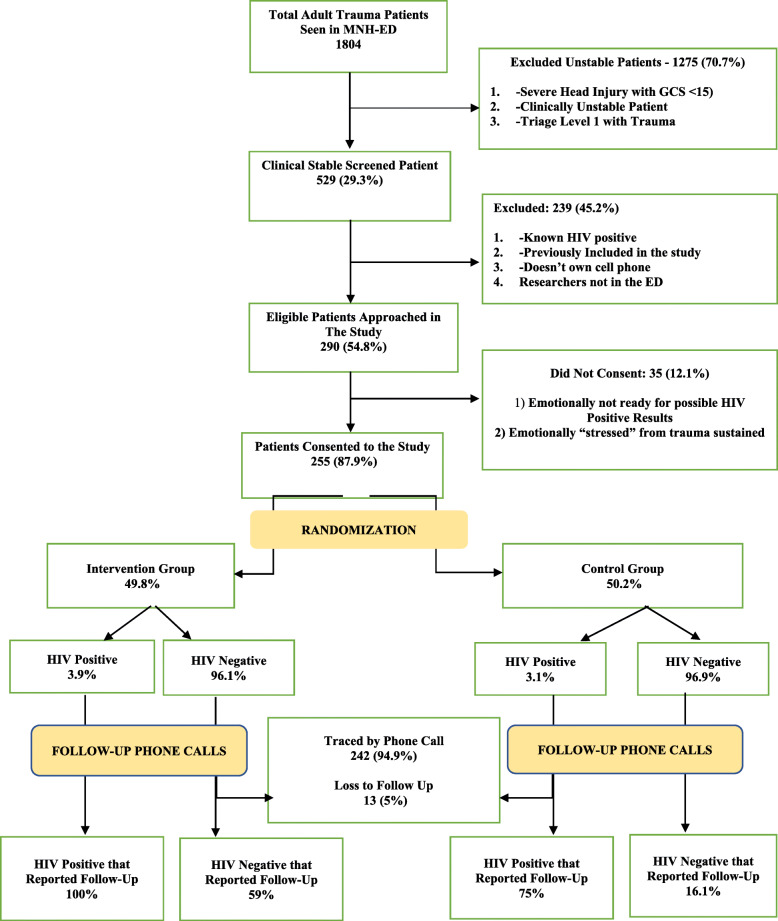
Participant flowchart

During the study period, 1804 adult trauma patients were seen in the MNH-ED. Of these patients, 70.7% were excluded due to severe injuries with instability (Fig. 1). The remaining were consecutively screened (*N* = 529), and 290 met the eligibility criteria. Of these, 255 (87.9%) consented to taking an HIV test and participating in the study. The reason for not participating was most commonly stress from the traumatic experience of being involved in an accident and not being emotionally ready to take on more stress if the results of the HIV test were positive. Of the 255 enrolled patients, we achieved phone follow-up for 242 (94.8%) patients.

Most participants were male (83%), and most participants were young adults with a median age of 29 years with an IQR of 24––40 years (Table 1). Approximately half were married; the majority (82.4%) had achieved primary education as their highest educational level. Most (82.4%) of the study population was referred from outside hospitals, and 85% of the study population had no health care insurance. The majority of participants were employed by others (i.e., hired staff, 60.4%). Of the 255 enrolled, 9 (6%) tested HIV-positive.

**Table 1:** Demographic characteristics of the study population

Demographic characteristic	Total, ***N*** = 255	Control group, ***n*** (%), ***N*** = 128	Intervention group, ***n*** (%), ***N*** = 127
**Sex**			
Male	211	103 (48.8%)	108 (51.2%)
Female	44	25 (56.8%)	19 (43.2%)
**Age groups**			
18–35 years	175	92 (52.6%)	83 (47.4%)
36–55 years	64	32 (50.0%)	32 (50.0%)
> 55 years	16	4 (25.0%)	12 (75.0%)
**Marital status**			
Divorced	11	5 (45.5%)	6 (54.5%)
Married	144	73 (50.7%)	71 (49.3%)
Single	97	49 (50.5%)	48 (49.5%)
Widowed	3	1 (33.3%)	2 (66.7%)
**Level of education**			
College	27	12 (44.4%)	15 (55.6%)
Informal	10	4 (40.0%)	6 (60.0%)
Primary	139	69 (49.6%)	70 (50.4%)
Secondary	74	39 (52.7%)	35 (47.3%)
University	5	4 (80.0%)	1 (20.0%)
**Occupation**			
Employed	154	72 (46.8%)	82 (53.2%)
Student	5	1 (20.0%)	4 (80.0%)
Unemployed	89	52 (58.4%)	37 (41.6%)
Unskilled Labor	7	3 (42.9%)	4 (57.1%)
**Insurance status**			
Insured	38	16 (42.1%)	22 (57.9%)
Non-insured	217	112 (51.6%)	105 (48.4%)
**Referral status**			
Referral	210	105 (50.0%)	105 (50.0%)
Non-referral	45	23 (51.1%)	22 (48.9%)
**HIV status**			
Negative	246	124 (50.4%)	122 (49.6%)
Positive	9	4 (44.4%)	5 (55.6%)

There were 128 (50.2%) participants in the control group and 127 (49.8%) participants in the intervention group (Fig. 1). There were more males in the intervention group and more females in the control group. More of the elderly participants were randomized into the intervention arm (Table 1). There were 5 HIV-positive and 122-HIV negative patients in the intervention group and 4 HIV-positive and 124 HIV-negative patients in the control group.

A review of the report from Extrateq™ showed that 381 text messages were successfully sent, consistent with 3 calls per person in the intervention group.

### Compliance with follow-up for recommended care

A total of 242 (94.9%) participants were reached by phone in follow-up. Among the 13 (5%) that were lost to follow-up, three were in the intervention group and ten were in the control group. All interventional group participants that were reached reported that they had received the text message at least once and all individuals in the control group denied having received any messages.

Among the 242 reached, 100 (41.3%) participants reported they completed follow-up care according to their respective HIV status; 77% of those who completed care were in the intervention group and 23% were in the control group (*P* = < 0.0001)**.** Relative risk (RR) for attending follow-up care was 3.4 (CI 2.3–5.0) if the patient had received a text. The number of patients needed to treat (NNT) (i.e., receive a text) to obtain one follow-up visit was 2.3 (95% CI 1.9–3.1) (Table 2).

**Table 2:** Follow-up visits according to treatment arm

	Number	Total follow up services in all study patients, *N* (%)	*P* value
Intervention group	127	77(60.6%)	< 0.0001
Control group	128	23(17.9%)	
*RR 3.4 (CI 2.3–5.0), NNT 2.3 (95% CI 3.1–1.9)			
	*N*	Repeated HIV screening in HIV negatives, *N* (%)	*P* value
Intervention group	122	72 (59.0%)	< 0.0001
Control group	124	20(16.1%)	
*RR 3.7 (CI 2.4–5.6), NNT 2.3 (95% CI 3.1–1.9)			
	*N*	Follow up visit to CTC in HIV positives, *N* (%)	Fisher exact probability
Intervention group	5	5 (100%)	N/A
Control group	4	3 (75%)	

### Follow-up by HIV status

In the HIV-negative population, 37.4% of patients underwent repeat screening in the recommended time period. Repeat screening was significantly more frequent in the intervention group compared with the control group (59.0% vs 16.1%, respectively, *P* < 0.0001). Sending a text message increased the likelihood of follow-up in HIV-negative patients with a RR = 3.65 compared to no text, and NNT was 2.3 (95% CI 1.9–3.1) (Table 2).

All 9 HIV-positive patients were reached by the study investigators. Among these, 8 (88.9%) reported having attended an initial CTC visit. All 5 in the intervention group and 3 out of 4 in the control group had attended an initial CTC visit. The numbers were too small for statistical analysis (Table 2).

## Discussion

This RTC tested the feasibility and efficacy of text messaging to promote follow-up care among otherwise healthy trauma patients screened for HIV seen at an emergency department in a limited income country. We found that most patients approached were willing to be tested and receive SMS messages. Follow-up with recommended care was significantly greater in those who received the text messages compared to those who did not, RR = 3.4.

Previous studies evaluating the effect of SMS in the promotion of patient care to improve compliance with care in HIV and other chronic illnesses [7, 8, 11–17]. A 2017 systematic review by Daher et al. including 99 studies in Africa, Asia, Europe, and America assessed whether digital innovations were feasible, acceptable, and had a general impact on the promotion of care in HIV and other sexually transmitted diseases. The review concluded that these innovations were feasible, acceptable, and had an impact on the promotion of care [13]. A 2017 meta-analysis by Fontelo et al., which analyzed 34 different studies globally, showed that text message was a valuable tool to increase general HIV-related compliance for clinic attendance and medication adherence [14].

Our study had several unique aspects. While prior RCTs have been conducted using digital interventions like text messages to improve the care of HIV patients in low- and middle-income countries (LMICs) [7, 8, 10, 15], most of these studies were confined to patients known to have or suspected to have HIV due to symptoms. While our study assessed the value of this intervention in asymptomatic patients who had not sought out HIV screening. This study also included HIV-negative patients and found that SMS significantly improved compliance with repeat testing at three months. A Kenyan study by Mugo et al. [8] comparing the impact of text messages, phone calls, and in-person appointment reminders on the rate of repeat screening of HIV also found that text messages increased the likelihood of accessing follow-up services. However, that study included only patients who had presented to a clinic because of their medical symptoms.

Another unique aspect of our study was the use of pre-programmed, automated SMS which allows messages to be sent to specific individuals at specified times. The intervention does not require having someone to remember to send the messages. The intervention had high fidelity: 381 messages were logged as sent, participants in the intervention arm remembered receiving a message, and no patients in the control arm reported receiving the SMS.

This study also shows the importance of provider-initiated counseling and testing for every patient entering the hospital. Among patients without medical complaints, and who would have not presented to a medical facility other than for trauma, we found a HIV incidence of 3.5%. This is substantially higher than in many ED-based screening programs in high-income countries. While we only included patients who were willing to be tested for HIV, the cohort study by Ramadhan et al. [5] at our emergency department enrolled participants first and then asked if they were willing to be tested and receive their results. In that study, 250 (76.7%) patients accepted testing for HIV, and among them, 98.8% were ready to receive their test results when asked during counseling, demonstrating again the willingness of people who are asymptomatic to be tested.

The success of this intervention in Tanzania can be explained by the wide availability of mobile phones in our and other limited and middle-income countries which allows communication even in remote areas. The use of SMS (as opposed to email) meant that patients did not need to have internet access or incur any cost to receive the messages, eliminating the burden of buying internet data packages.

A total of $20,000 was used in research tool development and $170 used for domain name registration, sending messages, and hosting the research app online. The number needed to treat suggests this is a small cost compared to the cost incurred by the individual and government to treat advanced HIV/AIDS and its opportunistic infections. Used broadly, this type of technology would have a significant impact on the transmission and treatment of HIV.

The overall rate of follow-up for care in our cohort was 39.2%, which is suboptimal. This shows the need for some type of intervention to improve compliance with care. Follow-up was significantly higher in the intervention group compared to the control group. Most of the patients were HIV-negative and were much more likely to follow-up for repeat screening if they received the SMS than if they were in the control group. HIV-positive patients had a good follow-up in both groups, but with only 9 people in total, the uncertainty surrounding the impact of the intervention is quite large. We would recommend that the Ministry of Health, Community Development, Gender, Elderly and Children plus the Government, and other stakeholders consider employing and funding SMS-based innovation to remind patients of important follow-up visits, not only in HIV but other chronic diseases, too.

### Study limitations and mitigation

Our outcome of follow-up with recommended care was based on self-report. Self-reporting can be affected by social desirability bias. However, we would not expect the intervention group to be more likely to provide positive answers than the control group. Secondly, this was a single-center study; our results may not be generalizable to other hospitals in Tanzania. However, Muhimbili National Hospital is a tertiary-level referral hospital, receiving patients from all over the country. Finally, the small number of HIV-positive participants prohibited useful statistical testing on the effect of the intervention in this group.

In conclusion, text messaging is a feasible, efficient, and effective way to increase follow-up visits among individuals tested for HIV in LMICs. This study was able to show a significant difference between patients that receive a reminder text and those that do not. This relatively low-cost method can help in decreasing the burden of disease from HIV as well as other chronic illnesses in LMICs.

## Data Availability

The dataset supporting this study is available from the authors on request.
